# Giant Adrenal Myelolipoma in a Patient without Endocrine Disorder: A Case Report and a Review of the Literature

**DOI:** 10.1155/2018/4854368

**Published:** 2018-06-11

**Authors:** Yoshifumi Nakayama, Nobutaka Matayoshi, Masaki Akiyama, Yusuke Sawatsubashi, Jun Nagata, Masanori Hisaoka, Keiji Hirata

**Affiliations:** ^1^Department of Surgery 1, University of Occupational and Environmental Health, 1-1 Iseigaoka, Yahata-nishi-ku, Kitakyushu 807-8555, Japan; ^2^Department of Gastroenterological and General Surgery, Wakamatsu Hospital of University of Occupational and Environmental Health, 1-17-1 Hamamachi, Wakamatsu-ku, Kitakyushu 808-0024, Japan; ^3^Department of Pathology and Oncology, School of Medicine, University of Occupational and Environmental Health, 1-1 Iseigaoka, Yahata-nishi-ku, Kitakyushu 807-8555, Japan

## Abstract

We herein present a surgically treated case of huge adrenal myelolipoma. A 62-year-old woman presented to our surgical outpatient clinic with a retroperitoneal tumor. A clinical examination revealed an elastic soft, smooth-surfaced, painless, child-head-sized tumor with poor mobility, which was located in the left upper abdomen. Computed tomography (CT) and magnetic resonance imaging (MRI) of the abdomen revealed an uneven tumor surrounding the stomach, spleen, pancreas, and left kidney, which was 20 × 18 × 10 cm in size. The retroperitoneal tumor was resected. The tumor was attached to the surrounding organs, including the pancreas, spleen, and left kidney, but had not directly invaded these organs. The tumor was yellow and elastic soft and covered with a thin film. The origin of the tumor was suggested to be the left adrenal gland. The weight of the excised tumor was 1500 g. The histopathological diagnosis was adrenal myelolipoma. The patient had an uneventful recovery and was discharged from the hospital on the thirteenth day after the operation. She has been followed up in our outpatient clinic.

## 1. Introduction

Adrenal myelolipoma (AML) is a relatively rare benign tumor composed of mature adipose tissues and a variable amount of hematopoietic elements. The male-to-female ratio is 1 : 1. The incidence of AML is reported to be 0.08–0.4% at autopsy [1]. AMLs are nonfunctional tumors that are usually asymptomatic; however, they have been known to coexist with other endocrine disorders, such as Cushing's syndrome, congenital adrenal hyperplasia (CAH), Conn's syndrome, and pheochromocytoma [2–4]. Recently, AMLs have been reported in patients with CAH with increasing frequency. One study indicated that myelolipoma was detected in 4% of patients with CAH [5].

The largest AML (size, 31 × 24.5 × 11.5 cm; weight, 6000 g) in a patient without endocrine disorder was described by Akamatsu et al. [6], while the largest AML in a patient with CAH (size, 34 × 24 × 10.5 cm; weight, 5900 g) was described by Boudreaux et al. [7].

We herein report a relatively rare case of a giant AML of 1500 g in weight in a patient without endocrine disorder and discuss our analysis of the literature.

## 2. Case Report

**Figure 1:**
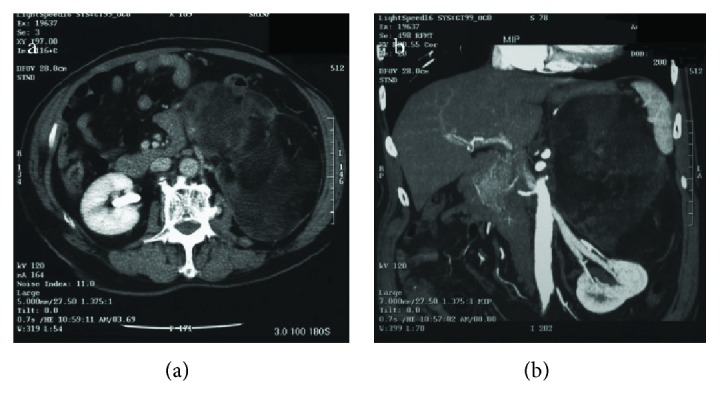
Abdominal computed tomography (CT) demonstrated a child-head-sized mass with heterogeneous contrast located in the left upper abdomen around the stomach, spleen, pancreas, and left kidney on the horizontal (a) and coronal (b) images.

A 62-year-old Japanese female patient presented with a left abdominal mass. She was referred to our surgical outpatient clinic to undergo a detailed examination and treatment for the left abdominal mass. A clinical examination revealed an elastic soft, smooth-surfaced, painless, child-head-sized tumor with poor mobility, which was located in the left upper abdomen. Abdominal computed tomography (CT) demonstrated a child-head-sized mass with heterogeneous contrast at the left upper abdomen around the stomach, spleen, pancreas, and left kidney on a horizontal image (Figure 1(a)) and coronal image (Figure 1(b)).

**Figure 2:**
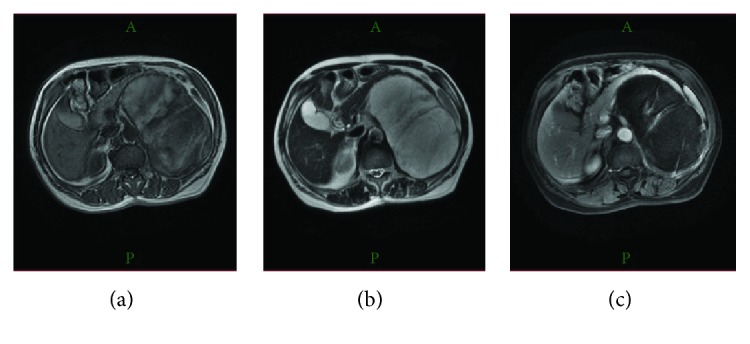
Magnetic resonance imaging (MRI) revealed a heterogeneously hyperintense mass on T1-weighted imaging (a), a relatively uniform and hyperintense mass on T2-weighted imaging (b), and a hypointense mass with an enhanced border on Gd-enhanced imaging (c).

Magnetic resonance imaging (MRI) revealed a heterogeneously hyperintense mass on T1-weighted imaging (Figure 2(a)), a relatively uniform and hyperintense mass on T2-weighted imaging (Figure 2(b)), and a hypointense mass with an enhanced border on gadolinium- (Gd-) enhanced imaging (Figure 2(c)). A retroperitoneal tumor was diagnosed. Her laboratory data were white blood cell count, 4600/mm^3^; hemoglobin, 12.8 g/dl; hematocrit, 36.5%; and platelet count, 182,000/mm^3^, with normal electrolytes, as well as normal blood urea nitrogen levels, but slight liver dysfunction. Her serum levels of corticosteroid and/or androgen were 13.3 ng/ml (10.4–35.0 in female) and 173 pg/dl (35.7–240.0), respectively, which are within the normal ranges; however, her serum level of ACTH was elevated at 138.70 pg/ml (7.2–63.3).

**Figure 3:**
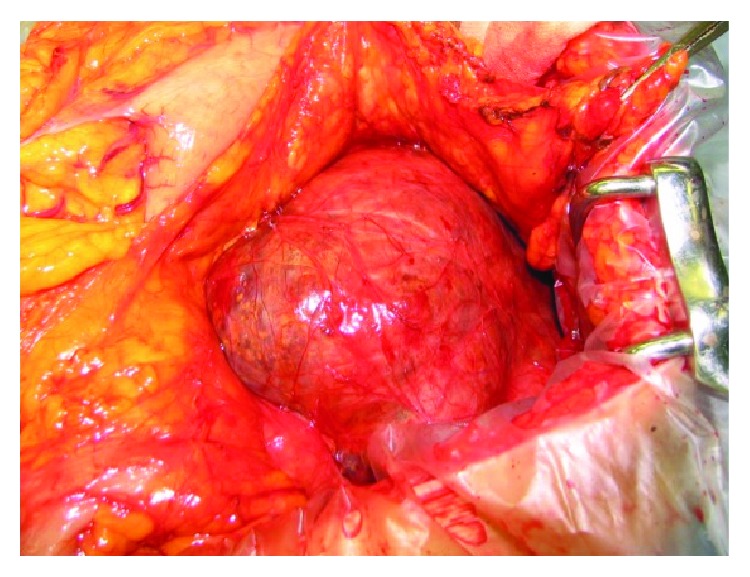
The operative findings revealed a yellow mass covered with a thin layer that was located at the left side of the stomach, posteriorly to the transverse mesocolon and pancreas, on the cranial side of the left kidney.

**Figure 4:**
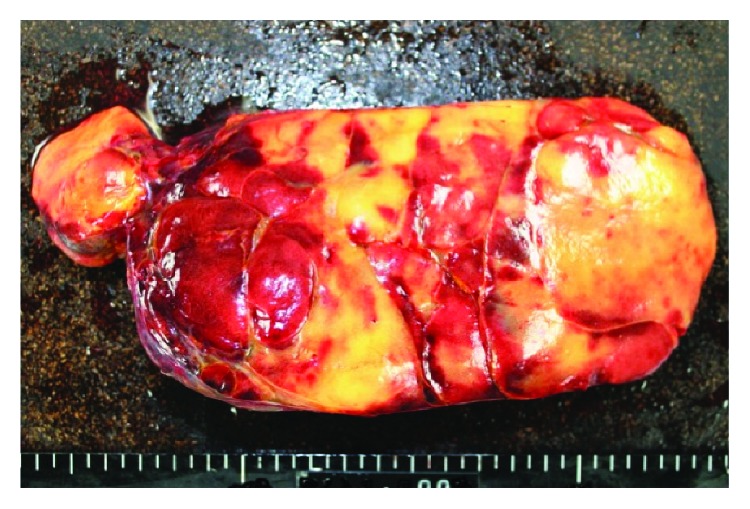
An examination of the cut surface of this tumor revealed a multilobular yellow mass with bleeding in places.

The retroperitoneal tumor was resected (Figure 3). The tumor was located at the left side of the stomach, posteriorly to the transverse mesocolon and pancreas, on the cranial side of the left kidney (Figures 1 and 2), but has not invaded the surrounding organs (Figures 1 and 2). The right adrenal gland was normal in size. The resected tumor was 20 × 18 × 10 cm in diameter and weighted 1500 g. An examination of the cut surface of the tumor revealed a multilobular yellow mass with bleeding in places (Figure 4).

**Figure 5:**
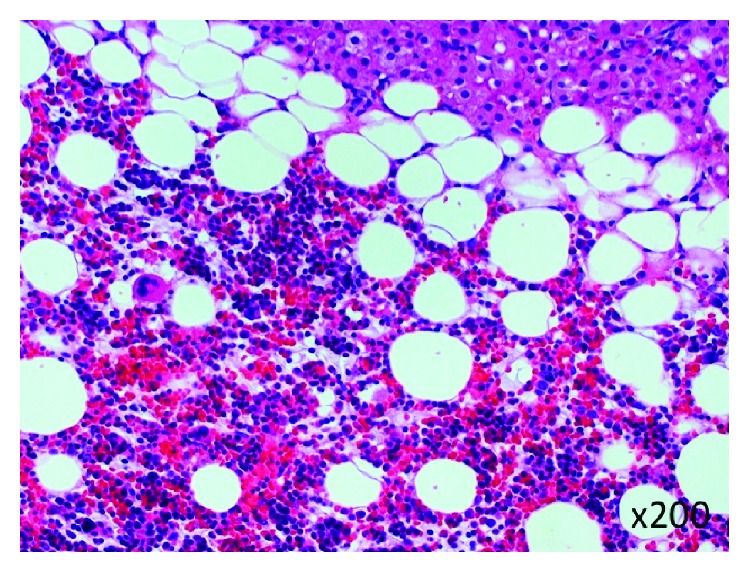
A histopathological examination (×200) with hematoxylin and eosin staining revealed that tumor was composed of a proliferation of mature and variable-sized adipocytes admixed with aggregates of hematopoietic elements.

A histopathological examination with hematoxylin and eosin staining revealed that the tumor was composed of a proliferation of mature and variable-sized adipocytes admixed with aggregates of hematopoietic elements, associated with adrenal gland tissue in the peripheral region within the tumor (Figure 5). These findings were compatible with AML.

The patient had an uneventful recovery and was discharged from the hospital on the 6th day after the operation. She has been followed up in our outpatient clinic without recurrence for approximately 12 years since undergoing the operation.

## 3. Discussion

The etiology of AML remains unclear. Some of the hypothesized etiologies include extramedullary hematopoiesis due to the autonomous proliferation of bone marrow cells transferred during embryogenesis, degeneration of epithelial tissues of the adrenal cortex, and adrenocortical cell metaplasia of the reticuloendothelial cells of the blood capillaries in response to stimuli such as necrosis, infection, or stress [1, 8–10]. The most widely accepted theory is that myelolipomas arise due to metaplasia of the reticuloendothelial cells of the blood capillaries in the adrenal gland in response to stimuli such as chronic stress, infection, necrosis, or inflammation [11, 12].

Although the diameter of AMLs ranges from less than 1 cm to more than 30 cm, they are usually less than 5 cm in diameter [13, 14]. AML is often asymptomatic, sometimes leading to very large adrenal masses (≧10 cm in diameter). These are often called “giant AML” [15]. Lawler et al. proposed a definition of the often quoted term “giant” AML [16]. We propose that giant AMLs of ≧1, 500 g should be called “real giant AMLs.” According to this criterion, we found the 21 cases involving giant AMLs in patients without endocrine disorders (Table 1) and the 6 cases involving giant AMLs in patients with CAH (Table 2).

Ultrasonography (US), computed tomography (CT), and magnetic resonance imaging (MRI) are effective for diagnosing AML in ≧90% of cases [4, 17]. Recently, with the widespread use of imaging studies such as US, CT, and MRI, the incidental detection of AML has been more common, and they now represent up to 10–15% of incidentally detected adrenal masses [18]. US shows myelolipoma as a well-defined tumor with varying degrees of hyperechoic (fatty tissue) and hypoechoic (myeloid tissue) components. CT shows myelolipoma as a well-delineated mass with heterogeneous attenuation and low-density fat tissue with more dense areas of myeloid tissue. MRI demonstrates myelolipoma as an area of high signal intensity on T1-weighted and T2-weighted sequences with reduced signal intensity on fat suppression and opposite phase imaging [18, 19].

Management of AML should be individualized. Small lesions, which are asymptomatic and measure less than 5 cm, should be monitored over a period of 1-2 years with imaging controls [20]. On the other hand, surgery is indicated when the patient is symptomatic, when the lesion is more than 5 cm in size due to rupture—which is a rare event—or when malignancy is suspected [20]. The most recognized complication of AML is spontaneous retroperitoneal hemorrhage [14, 16]. Daneshmand et al. suggested that symptomatic tumors or myelolipomas of ≧7 cm in size should be removed because they are associated with an increased risk of spontaneous rupture with retroperitoneal hemorrhage [4].

## 4. Conclusion

We reported a relatively rare case of a real giant AML that weighted 1500 g in a patient without an endocrine disorder. It is very important to provide suitable management on an individual basis.

## Figures and Tables

**Table 1:** Giant myelolipoma more than 1500 g without endocrine disorder.

Number	Author	Year	Gender	Age	Site	Size (cm)	Weight (g)	Symptoms	Ref. number
1	Akamatsu	2004	Male	51	Right	31 × 24.5 × 11.5	6000	Abd. mass, abd. pain	[6]
2	Wilhelmus	1981	Female	70	Left	30 × 22 × 16	5500	Abd. mass, abd. pain	[21]
3	Mukherjee	2010	Male	56	Right	28 × 26 × 17	5500	Abd. mass, weight loss	[22]
4	Kumar	2015	Male	40	Right	38 × 20 × 16	5200	Abd. pain, dyspnea, dizziness	[23]
5	Brogna	2011	Male	52	Left	25 × 20 × 20	4400	No	[24]
6	O'Daniel-Pierce	1996	Male	67	Right	30 × 20 × 11	4370	Abd. pain, abd. mass	[25]
7	Reshi	2007	Male	45	Right	25 × 14 × 11	>4000	Abd. mass	[26]
8	Gautam	2013	Male	52	Right	28 × 18 × 12	3850	Abd. pain, headache	[27]
9	Tanaka	1998	Male	50	Right	30 × 25 × 23	3500	Abd. mass	[28]
10	Dell'Avanzato	2009	Male	43	Right	22 × 18 × 9	3500	ND	[29]
11	Saha	2015	Female	59	Left	23 × 16 × 9	3300	Abd. distension, dragging sensation	[30]
12	Kumaresan	2011	Female	24	Right	30 × 20 × 18	3000	Abd. pain, abd. mass	[31]
13	Gerson	2015	Female	62	Right	21 × 18 × 9	2468	Abd. pain, nausea	[32]
14	Takahashi	2005	Male	48	Right	20 × 18 × 16	2400	Abd. distension, fever, diarrhea	[33]
15	Fernandes	2010	Male	48	Right	28 × 20 × 15	2200	Abd. pain, abd. mass	[34]
16	Chand	2017	Male	35	Right	24 × 15 × 12	2000	Pain in the right thigh	[35]
17	Répássy	2001	Female	50	Right	20 × 14	1650	Abd. pain, abd. discomfort	[36]
18	Andersom	2010	Man	35	Right	23.8 × 11.6 × 7.5	1575	Right-sided abd. discomfort	[37]
19	Goldman	1996	Male	42	Right	20.5 × 15 × 8.5	1550	Right frank pain, dizziness, vomiting	[38]
20	Ersoy	2006	Male	67	Right	12 × 10	1500	Abd. pain, fever	[39]
21	Chakrabarti	2012	Female	40	Right	15 × 10 × 8	1500	Abd. pain	[40]
22	Our case	2018	Female	62	Left	20 × 18 × 10	1500	Abd. mass	

ND: not described in abstract.

**Table 2:** Giant adrenal myelolipoma more than 1500 g with CAH.

Number	Author	Year	Gender	Age	Site	Size (cm)	Weight (g)	Symptoms	Ref. number
1	Boudreaux	1979	Male	56		34 × 24 × 10.5	5900	Dyspnea, low thoracic pain	[7]
2	McGeoch	2012	Male	34	Left	23 × 19 × 11	5800	Adb. mass	[41]
3	Kale	2015	Male	51	Left	34 × 20 × 13	4700	Back pain	[42]
4	Alvarez	2014	Female	44	Left	26 × 24 × 9.5	5090	Abd. pain, nausea, bilious emesis	[43]
5	Al-Bahri	2014	Male	39	Left, right	30 × 25 × 20 25 × 20 × 13	4100, 2700	Abd. distension, fatigue	[44]
6	German-Mena	2011	Male	45	Left	24.4 × 19.0 × 9.5	2557	Abd. distension, abd. pain	[45]

CAH: congenital adrenal hyperplasia.
